# The Evidence for Sparsentan-Mediated Inhibition of *I*_Na_ and *I*_K(erg)_: Possibly Unlinked to Its Antagonism of Angiotensin II or Endothelin Type a Receptor

**DOI:** 10.3390/biomedicines10010086

**Published:** 2021-12-31

**Authors:** Tzu-Hsien Chuang, Hsin-Yen Cho, Sheng-Nan Wu

**Affiliations:** 1 Department of Physiology, National Cheng Kung University Medical College, No. 1, University Road, Tainan 70101, Taiwan; s36091051@gs.ncku.edu.tw (T.-H.C.); s36094083@gs.ncku.edu.tw (H.-Y.C.); 2 Institute of Basic Medical Sciences, National Cheng Kung University Medical College, Tainan 70101, Taiwan

**Keywords:** sparsentan (4′-[(2-butyl-4-oxo-1,3-diazaspiro[4.4]non-1-en-3-yl)methyl]-*N*-(4,5-dimethyl-3-isoxazolyl)-2′-(ethoxymethyl)[1,1′-biphenyl]-2-sulfonamide), voltage-gated Na^+^ current, window Na^+^ current, resurgent Na^+^ current, *erg*-mediated K^+^ current, delayed-rectifier K^+^ current, current kinetics, pituitary cell, motor neuron-like cell

## Abstract

Sparsentan is viewed as a dual antagonist of endothelin type A (ET_A_) receptor and angiotensin II (AngII) receptor and it could be beneficial in patients with focal segmental glomerulosclerosis. Moreover, it could improve glomerular filtration rate and augment protective tissue remodeling in mouse models of focal segmental glomerulosclerosis. The ionic mechanisms through which it interacts with the magnitude and/or gating kinetics of ionic currents in excitable cells were not thoroughly investigated. Herein, we aimed to examine the effects of varying sparsentan concentrations on ionic currents residing in pituitary GH_3_ somatolactotrophs. From whole-cell current recordings made in GH_3_ cells, sparsentan (0.3–100 μM) differentially inhibited the peak and late components of voltage-gated Na^+^ current (*I*_Na_). The IC_50_ value of sparsentan required to exert a reduction in peak and late *I*_Na_ in GH_3_ cells was 15.04 and 1.21 μM, respectively; meanwhile, the K_D_ value estimated from its shortening in the slow component of *I*_Na_ inactivation time constant was 2.09 μM. The sparsentan (10 μM) presence did not change the overall current–voltage relationship of *I*_Na_; however, the steady-state inactivation curve of the current was shifted to more negative potential in its presence (10 μM), with no change in the gating charge of the curve. The window *I*_Na_ activated by a brief upsloping ramp was decreased during exposure to sparsentan (10 μM); moreover, recovery of peak *I*_Na_ became slowed in its presence. The Tefluthrin (Tef)-stimulated resurgent *I*_Na_ activated in response to abrupt depolarization followed by the descending ramp pulse was additionally attenuated by subsequent application of sparsentan. In continued presence of Tef (3 μM) or β-pompilidotoxin (3 μM), further application of sparsentan (3 μM) reversed their stimulation of *I*_Na_. However, sparsentan-induced inhibition of *I*_Na_ failed to be overcome by subsequent application of either endothelin 1 (1 μM) or angiotensin II (1 μM); moreover, in continued presence of endothelin (1 μM) or angiotensin II (1 μM), further addition of sparsentan (3 μM) effectively decreased peak *I*_Na_. Additionally, the application of sparsentan (3 μM) inhibited the peak and late components of *erg*-mediated K^+^ current in GH_3_ cells, although it mildly decreased the amplitude of delayed-rectifier K^+^ current. Altogether, this study provides a distinct yet unidentified finding that sparsentan may perturb the amplitude or gating of varying ionic currents in excitable cells.

## 1. Introduction

Sparsentan (4′-[(2-butyl-4-oxo-1,3-diazaspiro[4.4]non-1-en-3-yl)methyl]-*N*-(4,5-dimethyl-3-isoxazolyl)-2′-(ethoxymethyl)[1,1′-biphenyl]-2-sulfonamide), also known as RE-021, PS433540 and DARA-a, is a first-in-class, orally active, dual-acting, selective antagonist of the angiotensin II (AngII) type 1 receptor and the endothelin type A (ET_A_) receptor [1]. This compound has been in development for the treatment of focal segmental glomerulosclerosis [2,3,4,5,6,7,8,9,10,11]. It has been demonstrated that both endothelin and angiotensin II could injure podocytes inside the glomeruli [4,9,12] and that angiotensin II would exert the physiological (multiorgan) function, including ion channel-modifying effect [13]. Therefore, it is expected to provide meaningful clinical benefits in mitigating proteinuria as well as in improving glomerular filtration rate, although not specifically related to a cardiovascular therapy [7,8,11,12]. Notably, this compound could improve glomerular filtration rate and augment protective tissue remodeling in mouse models of focal segmental gloomerulosclerosis [14].

ET_A_ receptor transcripts have been previously reported to be identified in rat anterior pituitary [15]. The binding of endothelin to ET_A_ receptors in pituitary lactotrophs has been demonstrated to inhibit voltage-gated Ca^2+^ influx through G_i/o_ signaling pathway [16] and to decrease prolactin release [17]. Angiotensin II was also previously reported to influence the growth and angiogenic activity in pituitary GH_3_ cells [18]. Alternatively, endothelin-1 was previously reported to induce degeneration of cultured motor neurons [19] or to exercise action on the vestibular nuclei and on the maintenance of equilibrium [20]. However, at present, whether or how sparsentan can interact with these receptors to influence the functional activities of either pituitary cells or motor neurons has not been thoroughly investigated.

It has been established that the voltage-gated Na^+^ (Na_V_) channels contain the larger protein superfamily of voltage-gated ion channels and that their channel activity is key to the initiation, generation and propagation of action potentials in electrically excitable cells [21,22,23]. The Na_V_-channel family contains nine members which are denoted Na_V_1.1 through Na_V_1.9 [24]. These channels contain four internally homologous domains, referred to as DI to DIV, each of which consists of a six-helical transmembrane domain (S1–S6) and a reentry P loop residing between S5 and S6 [23,24,25]. Upon brief depolarization, Na_V_ channels readily go through a rapid transition from their resting (closed) state to the open state, and then to the inactivated state [22,23,24]. Several inhibitors or activators have been previously demonstrated to preferentially perturb the late component of voltage-gated Na^+^ current (*I*_Na_) [21,22]. However, the issue of how sparsentan could perturb the amplitude of kinetic gating of membrane ionic currents (e.g., voltage-gated Na^+^ current [*I*_Na_]) remains unmet. Alternatively, different types of K^+^ currents (e.g., delayed-rectifier K^+^ current [*I*_K(DR)_] and *erg*-mediated K^+^ current [*I*_K(erg)_]) might be regulated by binding to AngII or ET_A_ receptors [15,16,17,18,22]. Whether sparsentan, per se, might modify these ionic currents is also unclear.

In light of the above-mentioned initiatives, the electrophysiological effects of sparsentan and other functionally related compounds in pituitary GH_3_ somatolactotrophs were extensively studied in the present investigations. We sought to experimentally (1) determine whether the sparsentan presence has any effect on the amplitude and gating of *I*_Na_ residing in GH_3_ cells; (2) assess effects of sparsentan on resurgent *I*_Na_ (*I*_Na(R)_) or window *I*_Na_ (*I*_Na(W)_) stimulated by tefluthrin, a pyrethroid type-I insecticide; (3) study whether this compound has a significant influence on different types of K^+^ currents (e.g., *I*_K(erg)_ or *I*_K(DR)_). Findings from the present observations, for the first time, provide the evidence to show that the differential inhibition by sparsentan of peak and late *I*_Na_ is likely to be engaged in additional ionic mechanisms underlying its modifications on the functional activities of excitable cells (e.g., GH_3_ cells).

## 2. Materials and Methods

### 2.1. Chemicals, Drugs and Solutions Used in This Work

Sparsentan (RE-021, PS433540, PS-433540, DARA-a, BMS-346567, 4′-[(2-butyl-4-oxo-1,3-diazaspiro[4.4]non-1-en-3-yl)methyl]-*N*-(4,5-dimethyl-3-isoxazolyl)-2′-(ethoxymethyl)[1,1′-biphenyl]-2-sulfonamide, [1,1′-biphenyl]-2-sulfonamide, 2-[4-[(2-butyl-4-oxo-1,3-diazaspiro[4.4]non-1-en-3-yl)methyl]-2-(ethoxymethyl)phenyl]-*N*-(4,5-dimethyl-1,2-oxazol-3-yl)benzenesulfonamide, CAS number: 254740-64-2, C_32_H_40_N_4_O_5_S, PubChem CID: 10257882, https://pubchem.ncbi.nlm.nih.gov/compound/sparsentan (accessed on 14 November 2021) was acquired from Selleckchem (Asia Bioscience; Taipei, Taiwan). Angiotensin II (AngII), endothelin 1, tefluthrin (Tef), tetraethylammonium chloride (TEA) were supplied by Sigma-Aldrich (Merck, Taipei, Taiwan) and β-pompilidotoxin (Pomp) and tetrodotoxin (TTX) were by Alomone (Jerusalem, Israel). To protect sparsentan from light degradation, stock solution containing this compound was wrapped in aluminum foil.

Cell culture media, fetal bovine or calf serum, horse serum, L-glutamine and Trypsin-EDTA were supplied by HyCloneTM (Thermo Fisher, Genechain Industrial Co., Kaohsiung, Taiwan). Unless stated otherwise, other chemicals reagents were commercially available and of analytical grade.

The ion compositions of HEPES-buffered normal Tyrode’s solution used in this work were as follows (in mM): NaCl 136.5, KCl 5.4, CaCl_2_ 1.8, MgCl_2_ 0.53, glucose 5.5 and HEPES-NaOH buffer 5.5 (pH 7.4). For measurements of *I*_Na_ (i.e., peak and late *I*_Na_, *I*_Na(W)_ or *I*_Na(R)_), we kept GH_3_ cells immersed in Ca^2+^-free Tyrode’s solution in order to avoid the contamination by the magnitude of Ca^2+^-activated K^+^ currents and voltage-gated Ca^2+^ currents. To record K^+^ currents (*I*_K(DR)_ or *I*_K(erg)_), we filled up the recording electrode with a solution consisting of (in mM): K-aspartate 130, KCl 20, KH_2_PO_4_ 1, MgCl_2_ 1, Na_2_ATP 3, Na_2_GTP 0.1, EGTA 0.1, HEPES-NaOH 5 (pH 7.2), while to measure varying types of *I*_Na_, we substituted K^+^ ions in the internal solution for equimolar Cs^+^ ions and the pH in the solution was adjusted to 7.2 by adding CsOH. To record *I*_K(erg)_, the cells were bathed in a high-K^+^, Ca^2+^-free solution (in mM): KCl 130, NaCl 10, MgCl_2_ 3, glucose 6 and HEPES-KOH 5 (pH 7.4). All solutions used in this work were prepared using demineralized water from Milli-Q purification system (Merck). The bathing or filling solution and culture medium were filtered by using an Acrodisc^®^ syringe filter with Supor^®^ membrane (0.2 μm in pore size) (Bio-Check, Tainan, Taiwan).

### 2.2. Cell Preparations

Pituitary GH_3_ somatolactotrophs, supplied by the Bioresources Collection and Research Center ([BCRC-60015, https://catalog.bcrc.firdi.org.tw/BcrcContent?bid=60015 (accessed on 14 November 2021)]; Hsinchu, Taiwan), were grown in Ham’s F-12 medium supplemented with 15% heat-inactivated horse serum (*v*/*v*), 2.5% fetal calf serum (*v*/*v*) and 2 mM L-glutamine. Cells were grown in monolayer culture in 50 mL plastic culture flasks in a humidified environment of 5% CO_2_/95% air. Colorimetric method was commonly used in examining the densities of GH_3_ cells in microtiter plates by using an ELISA reader (Dynatech, Chantilly, VA, USA). The electrophysiological measurements were commonly carried out after cells reached confluence (usually 5–7 days).

### 2.3. Electrophysiological Measurements

During a few hours before the measurements, we dissociated GH_3_ cells with a 1% trypsin-EDTA solution, and a few drops of suspension containing single cells and clumps of cells were subsequently placed in a homemade chamber mounted on the stage of an inverted DM-IL fluorescence microscope (Leica; Major Instruments, Kaohsiung, Taiwan). Cells were bathed at room temperature (20–25 °C) in normal Tyrode’s solution, the composition of which was elaborated above, and they were allowed to attach to the chamber’s bottom before the recordings were made. The patch pipets were fabricated from Kimax-51 glass tubing with 1.5 mm outer diameter (#34500; Kimble, Dogger, New Taipei City, Taiwan) by using a vertical two-step puller (PP-83; Narishige, Major Instruments, Tainan, Taiwan). After being filled with the internal solution stated above, the recording electrodes had a tip resistance ranging between 3 and 5 MΩ; thereafter, during the measurements, they were firmly mounted in an air-tight holder, which had a suction port on the side, and chloride silver was used to be kept in contact with the internal electrode solution. We recorded ionic currents in the whole-cell configuration of a modified patch-clamp technique with the aid of either an Axoclamp-2B (Molecular Devices, Sunnyvale, CA, USA) or an RK-400 amplifier (Bio-Logic, Claix, France), as described elsewhere [26,27]. The liquid junction potentials, which emerged between the composition of the pipette solution differed from that in the bath, were zeroed shortly before formation of the seal, and the whole-cell data were then corrected.

The signals (e.g., current tracings) were consecutively monitored at a given interval and digitally stored online at 10 kHz in an ASUS ExpertBook laptop computer (P2451F; Yuan-Dai, Tainan, Taiwan). For efficient analog-to-digital (A/D) and digital-to-analog (D/A) conversion to proceed, a Digidata-1440A interfaced with the computer was operated by pClamp 10.6 software run on Microsoft Windows 7 (Redmond, WA, USA). The computer was placed on top of an adjustable Cookskin stand (Ningbo, China) to ensure efficient operation during the measurements.

### 2.4. Whole-Cell Data Analyses

To assess concentration-dependent inhibition of sparsentan on the peak (transient) or late amplitude of *I*_Na_ activated by a brief depolarizing pulse, we placed GH_3_ cells in Ca^2+^-free Tyrode’s solution which contained 10 mM TEA and 0.5 mM CdCl_2_. The examined cell was depolarized from a holding potential of −100 to 10 mV with a duration of 40 ms, and current amplitude was measured at the start (i.e., peak *I*_Na_) or end (i.e., late *I*_Na_) of each depolarizing pulse. Current amplitudes were measured and compared in the control period (i.e., sparsentan was not present) and during cell exposure to varying concentrations (0.3–100 μM) of sparsentan. The concentration (i.e., IC_50_) required to inhibit 50% of current amplitude (i.e., peak and late *I*_Na_) was determined according to the following first-order logistic sigmoidal equation (or a modified Hill equation):

$$Relative\;amplitude\;of\;I_{\mathrm{Na}}=\frac{\left(1-a\right)\times {\left[C\right]}^{-n_{H}}}{{\mathrm{IC}}_{50}^{-n_{H}}+{\left[C\right]}^{-n_{H}}}+a$$

where IC_50_ and n_H_ are the sparsentan concentration required for a 50% inhibition of peak or late *I*_Na_ and the Hill coefficient, respectively, and maximal inhibition (i.e., 1 − a) was also estimated from the equation.

The inhibitory effect of sparsentan on *I*_Na_ demonstrated here is thought to result from a state-dependent inhibitor that binds predominantly to the open state of the voltage-gated Na^+^ (Na_V_) channel. Judging from this simplifying assumption, we made a minimal binding scheme given as follows:

$$\mathrm{Closed}\overset{\alpha }{\underset{\beta }{⇄}}\;\mathrm{Open}\overset{k_{+1}{}^{*}\;\left[S\right]}{\underset{k_{-1}}{⇄}}\mathrm{Inactivated}$$

In this first-order scheme, [S] is the sparsentan concentration used and α and β are the voltage-gated rate constant for the opening and closing of the Na_V_ channel, respectively, while *k*_+1_* and *k*_−1_ represent the forward (i.e., on or bound) and backward (i.e., off or unbound) rate constant of sparsentan which interacts with the channel, respectively. Closed, open and inactivated in each term represent the closed (resting), open and inactivated state of the channel, respectively.

**Figure 1:**
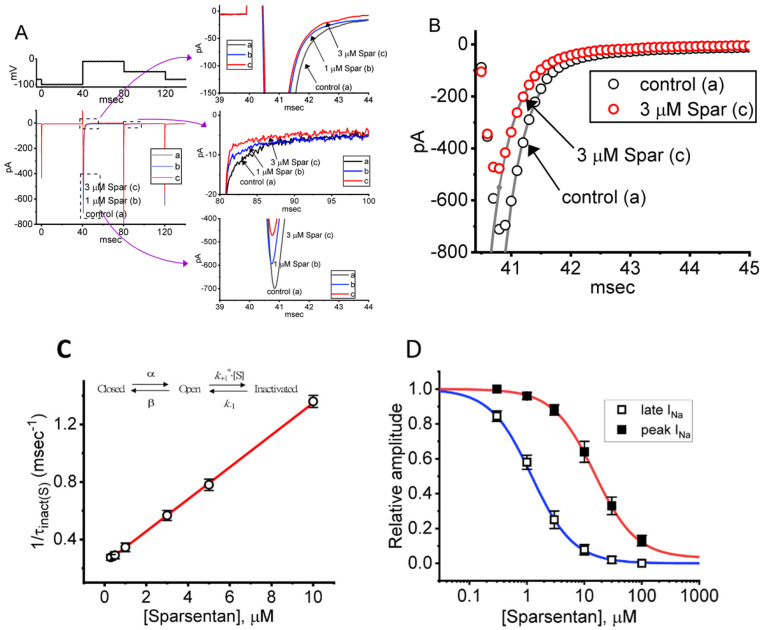
Effect of sparsentan (Spar) on voltage-gated Na^+^ current (*I*_Na_) recorded from pituitary GH_3_ cells. In this set of experiments, we placed cells in Ca^2+^-free Tyrode’s solution containing 10 mM tetraethylammonium chloride (TEA) and 0.5 mM CdCl_2_, and the recording pipettes were filled up with a Cs^+^-containing solution. Under voltage-clamp conditions (as indicated in top panel), the *I*_Na_ was evoked. (**A**) Representative current traces achieved in the control period (a, sparsentan was not present), and during cell exposure to 1 μM sparsentan (b) or 3 μM sparsentan (c). Current traces in the right upper, middle or lower panel (indicated on a faster time scale) demonstrate the expanded records from dashed box in the left side of (**A**). a: control; b: 1 μM sparsentan; c: 3 μM sparsentan. (**B**) Trajectories of *I*_Na_ activated by brief step depolarization. Black circle represents the digitized trace in the control period (a), while red circle is one taken in the presence of 3 μM sparsentan (c). Current trajectory achieved in the absence (a) and presence (b) of 3 μM sparsentan was optimally fitted by single exponential function with 3.37 and 1.72 ms, respectively. (**C**) Positive linear relationship of the sparsentan concentration versus the rate constant in the slow component of *I*_Na_ inactivation. Inset at the top depicts the minimal reaction scheme used in this kinetic study. Such scheme was detailed in Materials and Methods. Based on the scheme, the k_+1_* (the forward (on) rate constant) and k_−1_ (the reverse (off) rate constant) values were estimated to be 0.1116 μM^−1^ms^−1^ and 0.2332 ms^−1^, respectively; hence, the resultant K_D_ (k_−1_/k_+1_*) value was 2.09 μM. (**D**) Concentration-dependent inhibition of sparsentan on *I*_Na_ amplitude measured at the start (filled squares) and end (open squares) of 30 ms depolarizing step to −10 mV from a holding potential of −100 mV (mean ± SEM; *n* = 8 for each data point). The sigmoidal lines overlaid onto the data points were appropriately fitted by the modified Hill equation, as detailed in Materials and Methods. The IC_50_ value achieved at the start (i.e., peak [transient] *I*_Na_) and end (i.e., late *I*_Na_) of 40 ms step depolarization was estimated to be 15.04 and 1.21 μM, respectively.

At this point, the value of k_+1_* or k_−1_ was quantitatively evaluated from the time constant (τ_inact(S)_) in the slow component of *I*_Na_ inactivation collected during cell exposure to varying sparsentan concentrations. These rate constants could be hence optimized by using the following equation, where the rate constant k_+_* is linearly proportional to 1/τ_inact(S)_:

$$1/\tau_{\mathrm{inact}\left(S\right)}=\left[S\right]\times k_{+1}{}^{*}+k_{-1}$$

in which *k*_+1_* and *k*_−1_, respectively, can be determined by the slope and from the ordinate intercept at [S] = 0 of the linear regression, as we interpolated the relation of the reciprocal time constant (1/τ_inact(S)_) versus the sparsentan concentration (i.e., [S]) (Figure 1C). A measure of the dissociation constant (K_D_), which is equal to the k_−1_ value divided by the value of *k*_+1_*, can thereafter be yielded.

The quasi-steady-state inactivation curve of peak *I*_Na_ achieved with or without the sparsentan application in GH_3_ cells was established and then reliably fit with a Boltzmann distribution (or the Fermi–Dirac distribution):

(1) $$\frac{I}{I_{max}}=\frac{1}{1+e^{\frac{\left(V-V_{\frac{1}{2}}\right)qF}{RT}}}$$

where *I_max_* is the maximal peak *I*_Na_ in the absence or presence of 10 μM sparsentan, *V* the conditioning potential in mV, *V*_1/2_ the half-maximal inactivation in the relationship of the curve in mV, *q* the apparent gating charge in *e* and *F*, *R* and *T* are the usual constants.

### 2.5. Curve-Fitting Procedures and Statistical Analyses Used in This Work

In this study, we performed curve fitting (linear or nonlinear) to the experimental results that we obtained with the goodness of fit by using varying methods, such as Microsoft “Solver” function built in Excel^TM^ run under Office 365^®^ (Microsoft) and OriginPro^®^ 2021 software (OriginLab; Scientific Formosa, Kaohsiung, Taiwan). The emerging data in this work are presented as the mean ± standard error of the mean (SEM), with the observation number in which the experimental results were collected. We initially performed the traditional Student’s *t*-test (paired or unpaired) followed by one-way analysis of variance (ANOVA) for statistical analyses. However, when the difference among different groups needed to be evaluated, we further implemented post hoc Duncan’s multiple-range comparisons. The analyses in this study were achieved using the SPSS 20 statistical package (AsiaAnalytics, Taipei, Taiwan). Differences were considered statistically significant when the *p*-value was below 0.05.

## 3. Results

### 3.1. Effect of Sparsentan on Voltage-Gated Na^+^ Current (I_Na_) Measured from Pituitary GH_3_ Cells

For the first stage of experiments, we placed cells in a Ca^2+^-free Tyrode’s solution containing 10 mM tetraethylammonium chloride (TEA) and 0.5 mM CdCl_2_. The presence of TEA and CdCl_2_ was used to block the magnitude of nonspecific K^+^ and Ca^2+^ currents, respectively; and the composition of normal Tyrode’s solution was stated in Materials and Methods. During the measurements, we filled up the electrodes by using the Cs^+^-containing solution. When the whole-cell configuration was established, we voltage-clamped the tested cell at the level of −80 mV, and to ensure complete recovery of *I*_Na_, the voltage preceding the depolarizing pulse was set at −100 mV with a duration of 40 ms; thereafter, for the activation of *I*_Na_, the 40 ms depolarizing voltage pulse from −100 to −10 mV followed by a return to −50 mV for another 40 ms was imposed on the cell. Upon this protocol, the *I*_Na_ with a rapid activation, inactivation and deactivation was robustly evoked [21,28]. Of interest, one minute after GH_3_ cells were constantly exposed to spansentan (1 or 3 μM), the peak amplitude of *I*_Na_ was promptly decreased and the inactivation time course of the current concurrently increased (Figure 1A,B). For example, in the presence of 3 μM sparsentan, the peak *I*_Na_ amplitude activated by rapid depolarizing pulse from −100 to −10 mV was reduced to 582 ± 24 pA (*n* = 8, *p* < 0.05) from a control value of 698 ± 37 pA (*n* = 8); meanwhile, the slow component of inactivation time constant of *I*_Na_ (τ_inact(S)_) was diminished from 3.38 ± 0.27 to 1.71 ± 0.11 ms (*n* = 8, *p* < 0.05) in the presence of 3 μM sparsentan. However, neither changes in the fast component of inactivation time constant of the current, nor those in time to peak of *I*_Na_ in response to brief step depolarization were observed during exposure to 3 μM sparsentan. Meanwhile, the existence of sparsentan decreased the deactivation time course of *I*_Na_ measured at −50 mV. One minute after washout of sparsentan, the peak amplitude and τ_inact(S)_ value was returned to 689 ± 35 pA and 3.29 ± 0.24 ms, respectively.

It needs to be stressed that increasing the sparsentan concentration not only resulted in the decreased amplitude in the peak *I*_Na_ but also caused an increase in the rate of *I*_Na_ inactivation in response to brief membrane depolarization. At this point, according to the first-order binding scheme (elaborated under Materials and Methods), the relationship between 1/τ_inact(S)_ and the sparsentan concentration turned out to be linear (Figure 1C). Consequently, the forward rate (k_+1_*) increased linearly with the sparsentan concentration, whereas the backward rate (k_−1_) was little affected by adding sparsentan. The forward (k_+1_*) and backward (k_−1_) rate constant was hence calculated to be 0.1116 μM^−1^ms^−1^ and 0.2332 ms^−1^, respectively; furthermore, the apparent dissociation constant (i.e., K_D_ = k_−1_/k_+1_*) for the preferential binding of sparsentan to the open state of the Na_V_ channels was yielded to be 2.09 μM.

The relationship between the sparsentan concentration and the peak (or transient) or late (or end-pulse) component of *I*_Na_ in response to abrupt depolarizing pulse was further analyzed and constructed in GH_3_ cells. In these experiments, each cell was depolarized from −100 to −10 mV with a duration of 40 ms and current amplitudes at the beginning or end of the depolarizing command voltage during exposure to varying concentration (0.3–100 μM) of sparsentan were measured. Data in Figure 1D showed that the addition of sparsentan resulted in a concentration-dependent decline in the peak or late (or end-pulse) *I*_Na_ activated by such short depolarizing pulse. The IC_50_ value for sparsentan-mediated inhibition of peak and late *I*_Na_ was measured to be 15.04 and 1.21 μM, respectively. The IC_50_ value for sparsentan-induced inhibition of late *I*_Na_ is nearly close to the K_D_ value (2.09 μM) estimated above. Our present results, therefore, reflect that sparsentan exerts an inhibitory action on *I*_Na_ in GH_3_ cells, and that the late component of *I*_Na_ was inhibited to a greater extent than the peak of the current at the same concentration.

### 3.2. Effect of Sparsentan on Current-Voltage (I-V) Relationship and Steady-State Inactivation Curve of Peak I_Na_ in GH_3_ Cells

**Figure 2:**
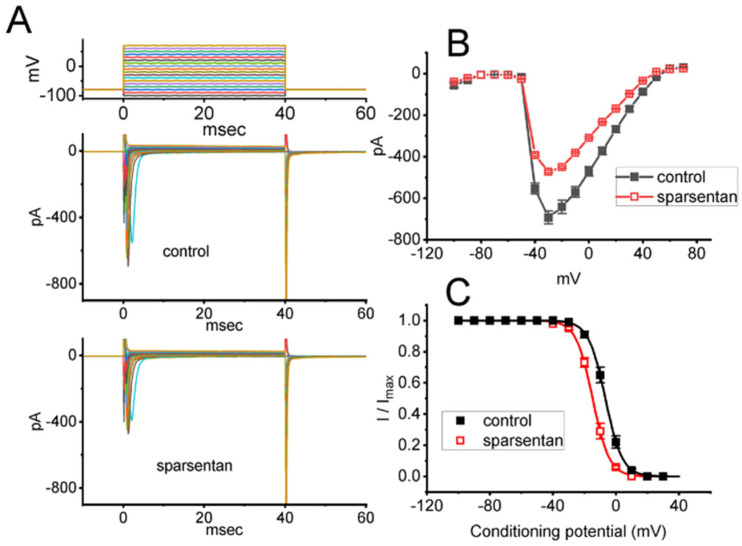
Effect of sparsentan on the current versus voltage (I-V) relationship (**A**,**B**) or inactivation curve of peak *I*_Na_ identified in GH_3_ cells. In these experiments, cells were bathed in Ca^2+^-free Tyrode’s solution, and we filled up the electrode with Cs^+^-containing solution. (**A**) Representative current trances achieved in the control period (i.e., sparsentan was not present; top panel) or during exposure to 10 μM sparsentan (bottom panel). The uppermost panel indicates the voltage-clamp protocol applied. (**B**) Averaged I-V relationship of peak *I*_Na_ in the absence (solid squares) or presence (open squares) of 10 μM sparsentan (mean ± SEM; *n* = 8 for each point). (**C**) Steady-state inactivation curve (i.e., I/I_max_ versus the conditioning potential) of peak *I*_Na_ without (solid squares) or with (open squares) the application of 10 μM sparsentan (mean ± SEM; *n* = 8 for each point). In these experiments, as the tested cell was maintained at −80 mV, a test pulse to −10 mV, the conditioning voltage pulses with a duration of 40 ms to varying membrane potentials ranging between −100 and +30 mV were imposed to it. The sigmoidal smooth curves obtained in the absence or presence of 10 μM sparsentan were least-squares fit by a modified Boltzmann equation (detailed under Materials and Methods). Of note, there is a leftward shift along the voltage axis in the steady-state inactivation curve of the current during the presence of sparsentan, despite no concurrent change in the gating charge of the current.

We further studied the inhibitory effect of sparsentan on *I*_Na_ at different levels of membrane potentials, and an I-V relationship of peak *I*_Na_ without or with the sparsentan existence was established. As depicted in Figure 2A,B, the overall I-V relationship of peak *I*_Na_ was found to remain unchanged during exposure to 10 μM sparsentan. Furthermore, with a two-step voltage protocol, the inhibitory effect of sparsentan (10 μM) on the quasi-steady state inactivation curve of peak *I*_Na_ was further characterized (Figure 2C). In this set of experiments, a 40 ms conditioning pulse to various membrane potentials (ranging between −100 and +30 mV in 10 mV step) was delivered to precede the 40 ms test pulse to −10 mV from a holding potential of −80 mV. Under this experimental protocol, the relationship between the conditioning potentials and the normalized amplitude (i.e., I/I_max_) of peak *I*_Na_ achieved in the absence and presence of 10 μM sparsentan was constructed and appropriately fit to a Boltzmann type sigmoidal function (elaborated under Materials and Methods). In the control group (i.e., sparsentan was not present), the values of V_1/2_ and q (apparent gating charge) of the inactivation curve were −6.7 ± 0.9 mV and 4.7 ± 0.2 e (*n* = 8), respectively; however, during the exposure to 10 μM sparsentan, the V_1/2_ and q value of the curve were −14.8 ± 1.1 mV and 4.8 ± 0.2 e (*n* = 8), respectively. As such, cell exposure to sparsentan not only decreased the maximal conductance of peak *I*_Na,_ but also negatively shifted the inactivation curve by approximately 8 mV. Conversely, we failed to show any change in the gating charge of the inactivation curve in the presence of sparsentan. Therefore, the steady-state inactivation curve of peak *I*_Na_ in the existence of this compound was shifted to a hyperpolarizing potential along the voltage axis with no clear adjustment in the gating charge of the curve.

### 3.3. Slowing in Recovery from I_Na_ Inactivation Caused by Sparsentan in GH_3_ Cells

**Figure 3:**
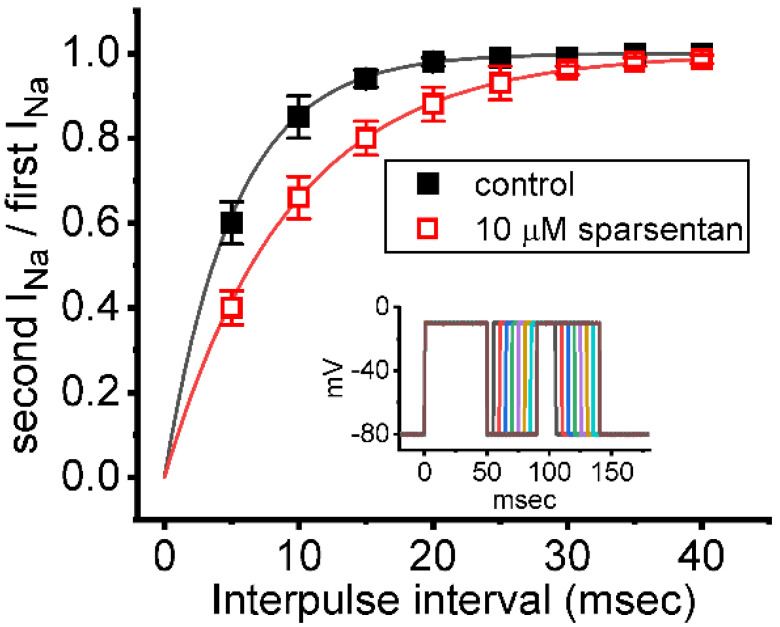
Effect of sparsentan on the recovery of *I*_Na_ block. In these experiments, as whole-cell configuration was firmly established, two-step voltage protocol was imposed on the examined cells. Inset indicates the voltage-clamp protocol applied. The relationship of interpulse pulse versus the relative amplitude achieved in the absence (solid squares) and presence (open squares) of 10 μM sparsentan (mean ± SEM; *n* = 8 for each point). The relative amplitude in the ordinate achieved obtained as the second amplitude activated by 50 ms depolarizing pulse from −80 to −10 mV was divided by the first one. The smooth curve in the absence and presence of 10 μM sparsentan was fit to single exponential function with time constant of 5.2 and 9.3 ms, respectively. Of note, the existence of sparsentan can result in a prolongation of recovery in *I*_Na_ block.

We further examined whether the existence of sparsentan could alter the recovery from inactivation of *I*_Na_. In these experiments, we measured recovery from inactivation by using a standard gapped pulse protocol with a variable interpulse interval. The ratio of the magnitude of the second and first pulse peak *I*_Na_ (i.e., relative amplitude) evoked in response to 50 ms depolarizing pulse from −80 to −10 mV was used as an indication of the degree of recovery from current inactivation. Results in Figure 3 showed that, in the control (i.e., sparsentan was not present), the peak amplitude of *I*_Na_ was almost completely recovered from inactivation when the interpulse interval was set at 40 ms. Additionally, the time course of recovery from current inactivation achieved in the absence and presence of 10 μM sparsentan was least-squares fit to a single-exponential function with a time constant of 5.2 ± 0.4 ms (*n* = 7) and 9.3 ± 0.7 ms (*n* = 7), respectively. The experimental observations reflect that the presence of sparsentan causes significant prolongation in the recovery from inactivation of peak *I*_Na_ in GH_3_ cells.

### 3.4. Effect of Sparsentan on the Window Component of I_Na_ in Response to the Upsloping Ramp Pulse

**Figure 4:**
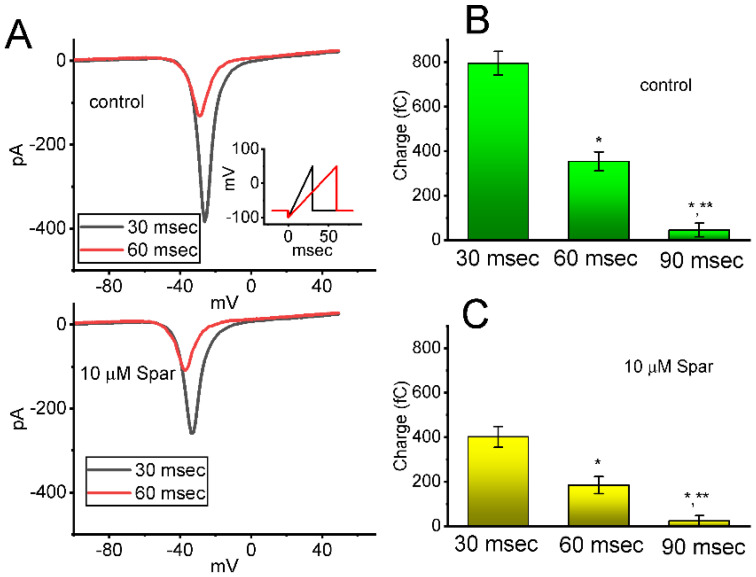
Effect of sparsentan (Spar) on window *I*_Na_ (*I*_Na(W)_) evoked by the upsloping ramp pulse. In these experiments, the examined cell was held at −80 mV, the ascending ramp pulse from −100 to +50 mV with a duration of 30 or 60 ms was imposed on it. (**A**) Representative current trance achieved by ramp pulse of a duration of 30 ms (black color) or 60 ms (red color) during the control period (top panel) or in the existence of 10 μM sparsentan (bottom panel). Inset in the right lower corner of panel (**A**) shows the voltage-clamp protocol used, while the downward deflection signifies inward current. The black or red color in each trace corresponds to that activated by voltage-clamp protocol shown in Inset. In (**B**,**C**), the total charges of instantaneous *I*_Na(W)_ evoked by the upsloping ramp pulse with different duration (30, 60 or 90 ms) were, respectively, demonstrated in the absence and presence of 10 μM sparsentan (mean ± SEM; *n* = 7 for each bar). Statistical analyses in (**B**,**C**) were performed by one-way ANOVA (*p* < 0.05). * Significantly different from those taken with the duration of 30 ms (*p* < 0.5), and ** significantly different from those with the duration of 60 ms (*p* < 0.05).

Next, whether the instantaneous window *I*_Na_ (*I*_Na(W)_) evoked by the ascending ramp pulse was able to be modified by the presence of sparsentan was further investigated in GH_3_ cells. In an effort to perform these experiments, the tested cell was maintained at −80 mV and the descending voltage pulse from −100 to +50 mV with a duration of 30 or 60 ms was thereafter imposed on it. As shown in Figure 4A, as cells were exposed to sparsentan (10 μM), the amplitude of *I*_Na(W)_ achieved by the downsloping ramp with a duration of 30 or 60 ms was substantially reduced. For example, the presence of 10 μM sparsentan decreased the peak amplitude of *I*_Na(W)_ activated by 30 ms ascending pulse from 389 ± 28 to 258 ± 22 pA (*n* = 8, *p* < 0.05). After washout of sparsentan, current amplitude was returned to 385 ± 25 pA (*n* = 7). The summary bar graph shown in Figure 4B,C depicts the total charges collected in response to the upsloping ramp pulse (from −100 to +50 mV) with a duration of 30 or 60 ms in the absence and presence of 10 μM sparsentan, respectively. Therefore, the results demonstrate that the amplitude or total charge of *I*_Na(W)_ evoked by abrupt ascending ramp voltage can evidently be reduced in the presence of sparsentan.

### 3.5. Effect of Tefluthrin (Tef) and Tef plus Sparsentan on Ramp-Evoked Resurgent _INa_ (I_Na(R)_) in GH_3_ Cells

**Figure 5:**
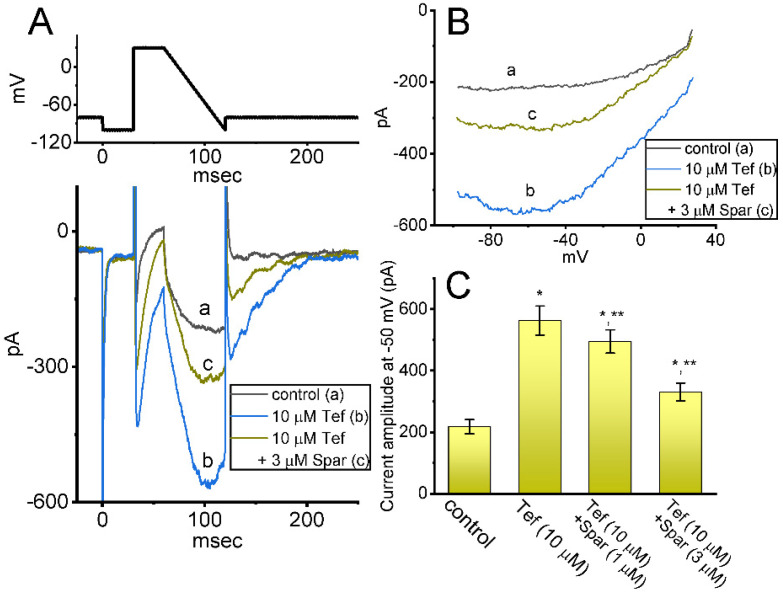
Effect of sparsentan (Spar) on resurgent *I*_Na_ (*I*_Na(R)_) identified in GH_3_ cells. These experiments were conducted in cells bathed in Ca^2+^-free Tyrode’s solution, and we filled up the electrode with Cs^+^-enriched solution. The examined cell was held at −80 mV, and to ensure complete recovery of *I*_Na_, the voltage preceding depolarizing pulse to +30 mV was set at −100 mV for 30 ms; thereafter, a descending ramp pulse from +30 to −100 ms with a duration of 60 ms was applied to evoke *I*_Na(R)_. (**A**) Representative current traces obtained in the control period (a) and during cell exposure to 10 μM tefluthrin (b, Tef) or to 10 μM Tef plus 3 μM sparsentan. The top panel shows the voltage-clamp protocol imposed, and downward deflections signify occurrence of inward current. (**B**) Relationship of instantaneous *I*_Na(R)_ versus membrane potential. a: control; b: 10 μM tefluthrin; c: 10 μM tefluthrin plus 3 μM sparsentan. (**C**) Summary bar graph showing effect of tefluthrin and tefluthrin plus sparsentan (1 or 3 μM) on the amplitude of *I*_Na(R)_ in GH_3_ cells (mean ± SEM; *n* = 7 for each bar). *I*_Na(R)_ amplitude activated by the 60 ms downsloping ramp pulse from +30 to −100 mV was measured at the level of −50 mV. Statistical analysis was made by one-way ANOVA (*p* < 0.05). * Significantly different from control (*p* < 0.05) and ** significantly different from Tef (10 μM) alone group (*p* < 0.05). Of note, as cells were exposed to Tef, subsequent presence of sparsentan was able to Tef-stimulate *I*_Na(R)_ in these cells.

Earlier reports have demonstrated the existence of *I*_Na(R)_ in these lactotrophs which were sensitive to stimulation by Tef [28,29]. This type of *I*_Na_ was noticed to exhibit a nonmonotonic voltage dependency on membrane repolarization [28,30]. Hence, we started out examining whether the sparsentan addition can lead to any adjustments on Tef-stimulated *I*_Na(R)_ present in these cells. Interestingly, as demonstrated in Figure 5, the instantaneous *I*_Na(R)_ evoked in response to the 60 ms downsloping ramp pulse from +30 to −100 mV was evidently increased during cell exposure to 10 μM Tef alone; moreover, further addition of 1 or 3 μM sparsentan was noticed to decrease Tef-mediated stimulation of *I*_Na(R)_ evoked by the abrupt descending ramp voltage. For example, cell exposure to 10 μM Tef significantly increased the *I*_Na(R)_ amplitude measured at −50 mV from 218 ± 23 to 563 ± 47 pA (*n* = 7, *p* < 0.05). In continued presence of Tef, the *I*_Na(R)_ amplitude at the same level of voltage was further reduced to 331 ± 29 pA (*n* = 7, *p* < 0.05) during cell exposure to Tef (10 μM) plus sparsentan (3 μM). Therefore, the experimental observations presented herein reflected that the instantaneous *I*_Na(R)_ responding to the abrupt descending ramp pulse was sensitive to activation by Tef, as reported previously [28,29,30], and that the further application of sparsentan became capable of attenuating Tef-stimulated *I*_Na(R)_ in these cells.

### 3.6. Effect of Tef, β-Pompilidotoxin, Tef plus Sparsentan and β-Pompilidotoxin plus Sparsentan on I_Na_ Measured from GH_3_ Cells

**Figure 6:**
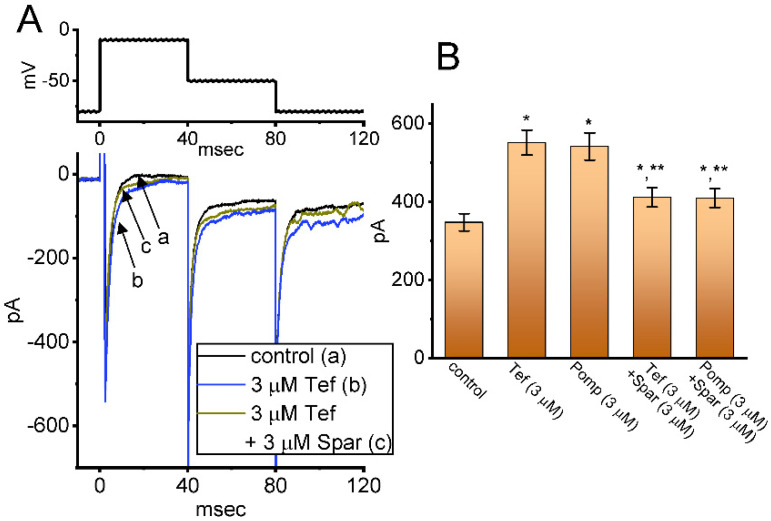
Effect of tefluthin, β-pompilidoxin, tefluthrin plus sparsentan, β-pompilidotoxin plus sparsentan on *I*_Na_ in GH_3_ cells. The voltage-clamp experiments were undertaken, as cells were voltage-clamped at −80 mV and the depolarizing pulse to −10 mV was imposed to them. (**A**) Representative current traces obtained in the control period (a, sparsentan was not present), and during exposure to 3 μM sparsentan (b) or to 3 μM sparsentan plus 3 μM tefluthrin (c). The top panel indicates the voltage-clamp protocol applied. Of note, the deactivating *I*_Na_ was additionally detected as membrane potential was returned to −50 mV with a duration of 40 ms. (**B**) Summary bar graph demonstrating effect of 3 μM tefluthrin, 3 μM β-pompilidotoxn, 3 μM tefluthrin plus 3 μM sparsentan and 3 μM β-pompilidotoxin plus 3 μM sparsentan on the peak amplitude of *I*_Na_ (mean ± SEM; *n* = 8 for each bar). The peak amplitude was measured at the start of 40 ms depolarizing command voltage from −80 to −10 mV. Tef: tefluthrin; Pomp: β-pompilidotoxin; Spar, sparsentan. Statistical analysis was performed by one-way ANOVA (*p* < 0.05). * Significantly different from controls (*p* < 0.05) and ** significantly different from Tef (3 μM) or Pomp (3 μM) alone group (*p* < 0.05).

We next investigated whether in continued exposure to tefluthrin or β-pompilidotoxin, subsequent application of sparsentan could lead to an attenuation in their stimulatory actions on *I*_Na_ in these cells. Tef is a type-I pyrethoid insecticide [31], while β-pompilidotoxin, a synthetic peptide, was originally isolated from Anoplius samariensis and Batozonellus maclifrons wasps [32]. Both of them have been reportedly viewed to increase *I*_Na_ as well as to exert considerable slowing in the time course of current inactivation [21,31,32,33]. As demonstrated in Figure 6, as GH_3_ cells were exposed to either 3 μM Tef or 3 μM β-pompilidotoxin, the *I*_Na_ activated by 40 ms depolarizing voltage step from −80 to −10 mV became evidently increased in combination with effective slowing in the inactivation time course of the current. Furthermore, in the continued presence of Tef or β-pompilidotxin, additional application of 3 μM sparsentan produced a substantial reduction in *I*_Na_, together with a robust shortening in the inactivation time constant of the current (Figure 6). For example, the existence of 3 μM β-pompilidotoxin increased peak *I*_Na_ amplitude from 348 ± 22 to 542 ± 35 pA (*n* = 8, *p* < 0.05); moreover, further application of 3 μM sparsentan significantly decreased peak current amplitude to 410 ± 24 pA (*n* = 8, *p* < 0.05). The results led us to reflect that, in GH_3_ cells, the addition of either Tef or β-pompilidotoxin can enhance *I*_Na_ magnitude and that subsequent application of sparsentan is effective at reversing their stimulatory actions on *I*_Na_.

### 3.7. Failure of Endothelin 1 or Angiotensin II to Alter Sparsentan-Indued Decrease in I_Na_

**Figure 7:**
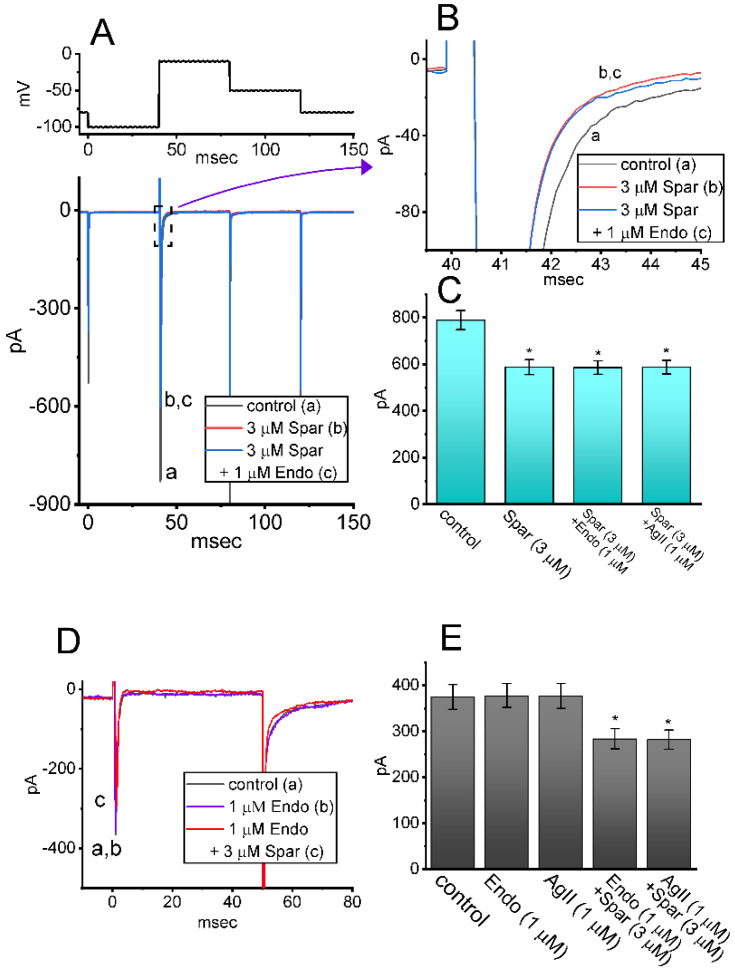
Effect of sparsentan, sparsentan plus endothelin 1 and sparsentan plus angiotensin II on *I*_Na_ in GH_3_ cells. The voltage-clamp experiments were performed in the tested cells, which were depolarized from −100 to −10 mV. (**A**) Representative current traces achieved in the control (a) and during exposure to 3 μM sparsentan (b) or to 3 μM sparsentan plus 1 μM endothelin 1. The upper part shows the voltage-clamp protocol used. In (**B**), current traces denote an expanded record from the dashed box in (A). a: control; b: sparsentan (3 μM); c: sparsentan (3 μM) plus endothelin 1 (1 μM). (**C**) Summary bar graph showing effect of sparsentan (3 μM), sparsentan (3 μM) plus endothelin 1 (1 μM) or sparsentan (3 μM) plus angiotensin II (1 μM) on the peak amplitude of *I*_Na_ (mean ± SEM; *n* = 7 for each bar). Current amplitude was measured at the beginning of depolarizing pulse from −100 to −10 mV. * Significantly different from control (*p* < 0.05). (**D**) Representative current traces taken in the control (a), and during exposure to 1 μM endothelin 1 (b) or 1 μM endothelin 1 plus 3 μM sparsentan (c). Current traces were evoked by depolarizing pulse from −80 to −10 mV with a duration of 50 ms. a: control; b: endothelin 1 (1 μM); c: endothelin 1 (1 μM) plus sparsentan (3 μM). (**E**) Summary bar graph showing effect of endothelin1 (1 μM), angiotensin II (1 μM), endothelin 1 (1 μM) plus sparsentan (3 μM), or angiotensin II (1 μM) plus sparsentan (3 μM) on peak *I*_Na_ (mean ± SEM; *n* = 7 for each bar). Current amplitude was measured at the beginning of depolarizing pulse from −80 to −10 mV. * Significantly different from control (*p* < 0.05). The statistical analyses in (**C**,**D**) were made by one-way ANOVA. Spar: sparsentan; Endo: endothelin 1; AgII: angiotensin II.

Sparsentan has been previously demonstrated to be a dual antagonist of endothelin type A (ET_A_) and angiotensin II (AngII) receptors [2,3,4,34]. The activity of ET_A_ or AngII receptors has been reported to be abundantly distributed in pituitary cells [15,18,35,36]. For these reasons, we explored whether sparsentan-mediated inhibition of *I*_Na_ found in GH_3_ cells could be altered by subsequent addition of endothelin 1 or angiotensin II. As shown in Figure 7A–C, the existence of 3 μM sparsentan resulted in a significant reduction in peak *I*_Na_ activated by short depolarizing voltage; however, further application of either endothelin 1 (1 μM) or angiotensin II (1 μM), still in the presence of sparsentan, failed to modify sparsentan-mediated reduction of peak *I*_Na_ in these cells. Furthermore, in the presence of endothelin 1 (1 μM) or angiotensin II (1 μM), subsequent addition of 3 μM sparsentan was effective at decreasing peak amplitude of *I*_Na_ (Figure 7D,E). It may be excluded, therefore, that sparsentan-mediated changes in the amplitude and gating of *I*_Na_ in response to short depolarizing pulse are largely associated with its antagonistic effect on either ET_A_ or AngII receptors, despite the existence of these receptors in pituitary cells [35,36].

### 3.8. Inhibitory Effect of Sparsentan on erg-Mediated K^+^ current (I_K(erg)_) Identified in GH_3_ Cells

**Figure 8:**
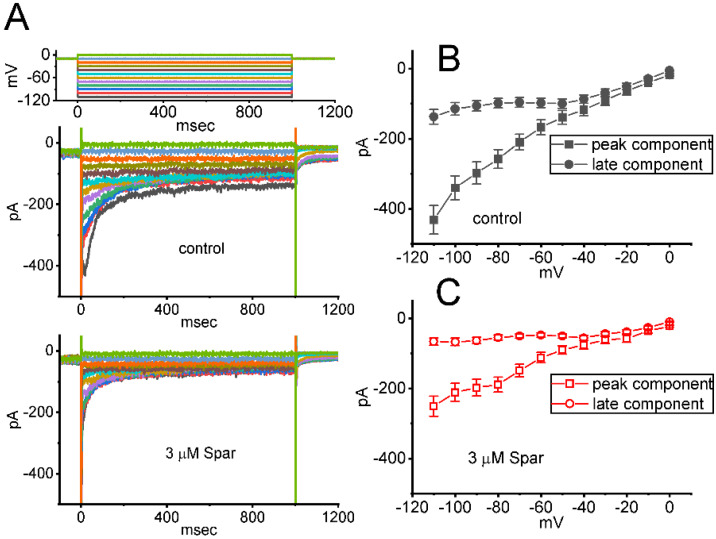
Effect of sparsentan (Spar) on erg-mediated K^+^ (*I*_K(erg)_) identified in GH3 cells. In this set of experiments, we bathed cells in high-K^+^, Ca^2+^-free solution containing 1 μM TTX and 0.5 mM CdCl_2_, and the recording pipette was filled with a K^+^-enriched solution. (**A**) Representative current traces evoked by the voltage-clamp protocol (indicated in the uppermost part). Current traces in top panel are controls and those in bottom panel were obtained in the presence of 3 μM sparsentan. The colors shown in current traces correspond to those in voltage-clamp protocol. In (**B**,**C**), averaged I-V relationships of the peak (square symbols) or late (circle symbols) component of *I*_K(erg)_ achieved in the control period (filled symbols) and during cell exposure to 3 μM sparsentan (open symbols), respectively. Each point in (**B**,**C**) indicates the mean ± SEM (*n* = 8 for each point). Statistical analyses in (**B**,**C**) were made by two way-ANOVA for repeated measures, P (factor 1, groups among data [peak versus sustained *I*_K(erg)_] taken at different level of membrane potentials) < 0.05, P (factor 2, groups between the absence and presence of sparsentan) < 0.05, P (interaction) < 0.05, followed post hoc Duncan’s multiple-range comparisons (*p* < 0.05).

In a separate set of whole-cell current recordings, different types of ionic currents (e.g., *I*_K(erg)_) were further examined with respect to possible effects of sparsentan on them. In these experiments, cells were bathed in high-K^+^, Ca^2+^-free solution containing 1 μM TTX and 0.5 CdCl_2_, while the recording electrode was filled up with K^+^-containing solution. The tested GH_3_ cells were voltage-clamped at −10 mV and varying voltage pulses ranging between −110 and 0 mV in 10 mV step with a duration of 1 sec were then delivered to them. Figure 8A illustrates the representative current traces obtained in the absence or presence of 3 μM sparsentan. The averaged I-V relationships of peak or late *I*_K(erg)_ taken from the control period (i.e., sparsentan was not present) and during cell exposure to 3 μM sparsentan were depicted in Figure 8B,C, respectively. For example, one minute after cell exposure to 3 μM sparsentan, the peak amplitude of deactivating *I*_K(erg)_ measured by the hyperpolarizing pulse from −10 to −110 mV was significantly decreased by 42 ± 3 % to 251 ± 29 pA (*n* = 8, *p* < 0.05) from a control value of 432 ± 41 pA (*n* = 8). After sparsentan was removed, current amplitude was returned to 428 ± 37 pA (*n* = 8). Therefore, the *I*_K(erg)_ inherently residing in GH_3_ cells can be sensitive to be blocked due to the existence of sparsentan.

### 3.9. Mild Inhibition of Delayed-Rectifier K^+^ Current (I_K(DR)_) Produced by Sparsentan in GH_3_ Cells

**Figure 9:**
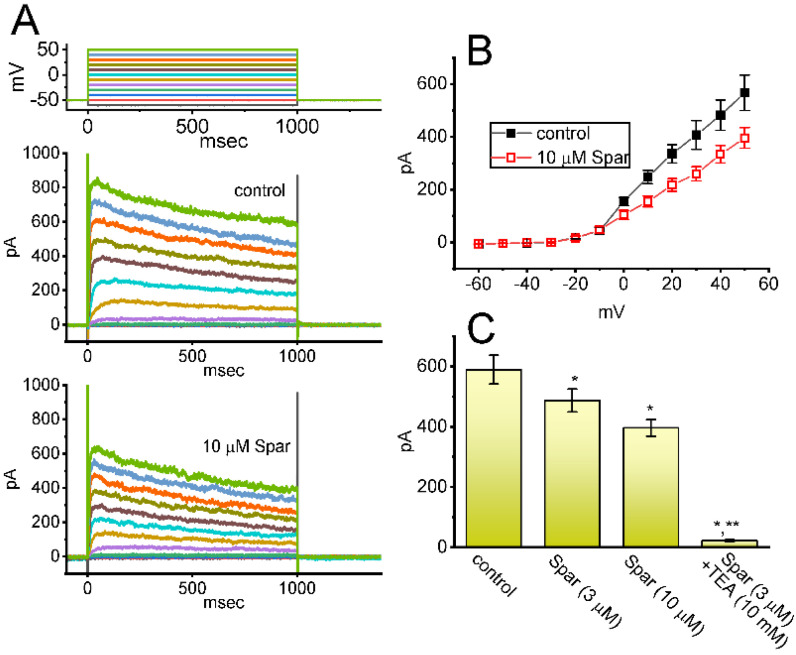
Effect of sparsentan (Spar) on delayed-rectifier K^+^ current (*I*_K(DR)_) recorded from GH_3_ cells. These experiments were performed in cells bathed in Ca^2+^-free Tyrode’s solution containing 1 μM TTX and 0.5 mM CdCl_2_, and the electrode was filled with K^+^-containing solution. (**A**) Representative current traces obtained in the control period (top panel) and during cell exposure to 10 μM sparsentan (bottom panel). The uppermost part shows the voltage-clamp protocol applied. (**B**) Averaged I-V relationships of *I*_K(DR)_ achieved in the absence (filled squares) and presence (open squares) of 10 μM sparsentan (mean ± SEM; *n* = 8 for each point). Current amplitude with or without sparsentan was achieved at the end of each voltage step. Statistical analyses were made by two way-ANOVA for repeated measures, P (factor 1, groups among data taken at different level of membrane potentials) < 0.05, P (factor 2, groups between the absence and presence of sparsentan) < 0.05, P (interaction) < 0.05, followed post hoc Duncan’s multiple-range comparisons (*p* < 0.05). (**C**) Summary bar graph showing effect of sparsentan (Spar, 3 and 10 μM) and sparsentan (10 μM) plus tetraethylammonium chloride (TEA, 10 mM) on the *I*_K(DR)_ amplitude at +50 mV. Each bar represents the mean ± SEM (*n* = 8). Statistical analysis was made by one-way ANOVA. * Significantly different from control (*p* < 0.05), and ** significantly different from sparsentan (3 μM) alone group (*p* < 0.05).

We further studied whether *I*_K(DR)_ activated by 1 s depolarizing pulse, another type of K^+^ current enriched in these cells, could be subject to any modifications by the presence of sparsentan. Cells were kept bathed in Ca^2+^-free Tyrode’s solution that contained 1 μM TTX and 0.5 mM CdCl_2_, and the electrode that we used was filled up with K^+^-containing solution. As demonstrated in Figure 9A,B, the *I*_K(DR)_ activated by depolarizing command voltages was slightly inhibited by the presence of 3 or 10 μM. The overall I-V relationship of *I*_K(DR)_ measured at the end of each voltage pulse was diminished during cell exposure to sparsentan at a concentration of 10 μM. For example, as the tested cells were depolarized from −50 to +50 mV with a duration of 1 sec, the presence of 10 μM sparsentan significantly decreased *I*_K(DR)_ amplitude by 30 ± 3 % from 567 ± 67 to 396 ± 39 pA (*n* = 8, *p* < 0.05). After washout of sparsentan, current amplitude was returned to 561 ± 63 pA (*n* = 7). However, no evident change in the inactivation time course of *I*_K(DR)_ was noticed during exposure to sparsentan. Moreover, as GH_3_ cells were continually exposed to 10 μM sparsentan, further addition of TEA (10 mM), an inhibitor of K^+^ channels, almost abolished the amplitude of *I*_K(DR)_ activated by the depolarizing pulse from −50 to +50 mV. In this instance, the results indicate that, by comparison, the presence of sparsentan (10 μM) exercises a mild but significant inhibitory effect on I_K(DR)_ amplitude measured from GH_3_ cells.

## 4. Discussion

In this study, the amplitude of *I*_Na_ recorded from GH_3_ cells noticeably decreased during cell exposure to sparsentan. Meanwhile, the inactivating or deactivating time course of the current became concurrently fastened by adding this compound, although the initial rising phase of *I*_Na_ (i.e., the activation time course) remained little affected. According to the estimated τ_inact(S)_ value of the current achieved in the presence of different sparsentan concentrations, the K_D_ value in GH_3_ cells was also yielded at 2.09 μM (Figure 1C). Of interest, this value was akin to the IC_50_ valued required for sparsentan-mediated inhibition of late *I*_Na_, but smaller than that for its suppression of peak *I*_Na_ (Figure 1D). The data suggest to us that these two results would be dovetailed together, despite probably an oversimplification of the binding scheme. Nonetheless, we found that sparsentan at the concentration range of 0.3–3 μM had almost no effect on the peak (or transient) component, while it effectively blocked late (end-pulse) *I*_Na_ measured at the end of depolarizing stimuli. There is hence a selective block of late *I*_Na_ by sparsentan.

It needs to be mentioned herein that, in the continued presence of tefluthrin or β-pompilidotoxin, the subsequent addition of sparsentan to the bath was able to reverse their stimulation of either *I*_Na(R)_ or peak and late *I*_Na_ observed in GH_3_ cells. During the exposure to sparsentan, the steady-state inactivation curve of peak *I*_Na_ was shifted in a hyperpolarizing direction with no modification in the gating charge of the current (Figure 2B); moreover, the recovery of *I*_Na_ block by responding to two-step voltage protocol was found to slow (Figure 3). Therefore, the experimental results revealed that sparsentan was capable of changing the amplitude and gating of *I*_Na_ identified in GH_3_ cells, suggesting that this compound may interact preferentially with the Na_V_ channels in the open state (or conformation) to perturb the magnitude and/or gating kinetics of voltage-activated *I*_Na_, including *I*_Na(R)_ and *I*_Na(W)_. Alternatively, the sparsentan molecule would be allowed to reach the blocking site only when the channel is in the open state. The mRNA transcripts for the α-subunit of Na_V_1.1, Na_V_1.2 and Na_V_1.6 were reported to be present in GH_3_ cells [22,37,38]. Whether sparsentan’s ability to alter either *I*_Na_ residing in heart cells (i.e., Na_V_1.5) or other isoforms of Na_V_ channel occurs is worthy of be further investigated.

Sparsentan was previously reported to be efficacious and long acting in the big endothelin 1-induced pressor model. Sparsentan caused a significant reduction in blood pressure at the lowest dose tested (10 μmol/kg/day) in spontaneously hypertensive rats. Sparsentan was previously reported to dose-dependently antagonize the angiotensin II-induced pressor response with an ED_50_ value of 0.8 μmol/kg (i.v.) and 3.6 μmol/kg (p.o.) [2]. This compound may hence exert the repurposing effects on *I*_Na_ or *I*_K(erg)_ demonstrated here and these action could be of therapeutic or clinical relevance.

One would expect that sparsentan-induced inhibition of *I*_Na_ detected in GH_3_ cells could be partly due to its inhibitory effect on the voltage-gated Ca^2+^ currents which are functionally expressed in excitable cells. However, under our experimental protocol, the voltage-activated inward currents were sensitive to stimulation by Tef or β-pompilidotoxin. Tef or β-pompilidotoxin has been broadly reported to be an activator of *I*_Na_ [21,28,31,32]. Moreover, in the continued presence of Tef or β-pompilidotoxin, additional exposure of cells to sparsentan was found to reverse the increase in *I*_Na(R)_ or peak *I*_Na_ stimulated by these compounds (Figure 5 and Figure 6). It is shown herein, hence, that the *I*_Na_ (i.e., late *I*_Na_, *I*_Na(R)_ and *I*_Na(W)_) in GH_3_ cells is highly susceptible to being blocked by sparsentan and that its block on late *I*_Na_ is actually larger than that on peak *I*_Na_. Thus, caution needs to be observed in avidly attributing the actions of sparsentan on cellular function to the inhibition of ET_A_ and AngII receptors. Moreover, the enhancement of late *I*_Na_ has been demonstrated to produce Ca^2+^ overload by decreasing the driving force for Ca^2+^ extrusion through the Na^+^-Ca^2+^ exchanging process [39]. Therefore, sparsentan-mediated inhibition of late *I*_Na_ would be responsible for the attenuation of cytosolic Ca^2+^ overload in excitable cells.

Sparsentan has been reportedly viewed to be a dual antagonist of ET_A_ and AngII receptors [2,3,4,34]. However, in this study, as GH_3_ cells were continually exposed to sparsentan, neither subsequent application of endothelin 1 nor angiotensin II was able to overcome sparsentan-mediated inhibition of *I*_Na_; moreover, in the presence of endothelin 1 or angiotensin II, further addition of sparsentan could suppress peak *I*_Na_ (Figure 7). The existence of sparsentan was additionally found to inhibit the amplitude of *I*_K(erg)_ and *I*_K(DR)_ in GH_3_ cells (Figure 8 and Figure 9). Under this scenario, sparsentan-perturbed adjustments in membrane ion currents demonstrated herein tend to be acute in onset, and they are mostly unlikely to be explained by its blockade of ET_A_ or AngII receptors. The concerted effectiveness of sparsentan in the antagonism of ET_A_ and AngII receptors, as well as direct inhibition of either *I*_Na_, including *I*_Na(R)_ and *I*_Na(W)_, or *I*_K(erg)_, may synergistically influence the functional activities of excitable cells (e.g., GH_3_ cells) occurring in vivo.

Altogether, the sparsentan’s effectiveness in modulating the amplitude and gating of *I*_Na_ demonstrated in this study led us to propose that this compound would become capable of exerting significant influence on electrical behaviors of the in vivo endocrine cells or neurons. However, sparsentan is regarded to be a highly potent dual ET_A_ and AngII receptor antagonist with K_i_s of 9.3 and 0.8 nM, respectively, which is noticeably lower than the IC_50_ and K_D_ value required for sparsentan-mediated inhibition of late *I*_Na_ and slowing in current inactivation, respectively. Alternatively, ranolazine, a blocker of late *I*_Na_ [39,40,41,42], has been noticeably reported to alleviate contrast-associated acute kidney injury as well as to dilate intrarenal arteries [43,44,45]. Sparsentan was also recently demonstrated to improve glomerular blood flow and to augment protective tissue remodeling in mouse models of focal segmental glomerulosclerosis [14]. Therefore, the extent to which its inhibitory actions on ionic currents (e.g., *I*_Na_ and *I*_K(erg)_) are intimately linked to the reduction in proteinuria in patients with focal segmental glomerulosclerosis remains to be further resolved, although Na^+^ and K^+^ ions do not appear to be osmotically active particles for determination of colloid osmotic pressure. While the detailed underlying mechanisms, by which sparsentan alters the strength of ionic currents, remain resolved, findings from this study provide novel insights into electrophysiological and pharmacological properties of sparsentan, which appear to be independent of its binding to ET_A_ or AngII receptors. The interaction of sparsentan with membrane ionic currents may, at least in part, be engaged in the underlying mechanisms through which it affects the functional activities of endocrine or neuronal cells. Whether sparsentan or other structural similar compounds (e.g., atrasentan and BMS-248360) exert any overarching effects on the magnitude and/or gating of *I*_Na_ or other types of ionic currents warrants further investigation.

## Data Availability

The original data is available upon reasonable request to the corresponding author.

## Abbreviations

ANOVA	analysis of variance
AngII	angiotensin II
ET_A_ receptor	endothelin type A receptor
*erg*	*ether-à-go-go*-related gene
*I-V*	current versus voltage
IC_50_	concentration required for half-maximal inhibition
*I*_K(DR)_	delayed-rectifier K^+^ current
*I*_K(erg)_	*erg*-mediated K^+^ current
*I*_Na_	voltage-gated Na^+^ current
*I*_Na(R)_	resurgent *I*_Na_
*I*_Na(W)_	window *I*_Na_
Na_V_ channel	voltage-gated Na^+^ channel
K_D_	dissociation constant
SEM	standard error of mean
TEA	tetraethylammonium chloride
Tef	tefluthrin
τ_inact(S)_	slow component of inactivation time course
TTX	tetrodotoxin
